# A new algorithm for aortic compliance evaluation in normals

**DOI:** 10.1186/1532-429X-11-S1-P284

**Published:** 2009-01-28

**Authors:** Yi Wang, Jianping Zhang, Edwin Estrada, Nathaniel Reichek

**Affiliations:** 1 St. Francis Hospital, Roslyn, NY USA; 2 Stony Brook University, Stony Brook, NY USA

**Keywords:** Phase Contrast, Pulse Wave Velocity, Phase Contrast Image, Aortic Pulse Wave Velocity, Aortic Compliance

## Introduction

Aortic compliance (AC) can be evaluated noninvasively and its reduction with age in normals has been demonstrated with both MRI and Doppler echo methods. Aortic pulse wave velocity (PWV), a measurement of the flow pulse traveling along aorta as a surrogate of AC, can be assessed using a single breath-hold phase contrast (PC) imaging technique. Accurate determination of the time delay (Δt) between flows in ascending and descending aortic regions is critical in PWV assessment. Various approaches have been studied for Δt, including measuring the intervals between flow onset points, between maximal flow points, and between parallel upslopes after least squares fittings. We studied a new approach using a sliding window maximal upslope approach.

## Methods

Eighty healthy volunteers with informed consent (age: 59.5 ± 13.9) were screened to exclude hypertension, elevated total cholesterol and cardiovascular disease. Using the 'candy cane' view of aorta, an axial plane through the ascending and descending aorta at the pulmonary artery level was prescribed and a through-plane velocity encoded PC cine imaging was acquired with VENC of 150 cm/s, TR/TE/FA = 98 ms/2.9 ms/15° and voxel spatial resolution 1.3 × 2 × 6 mm^3^ on a 1.5 T MRI scanner. The distance traveled by the aortic pulse wave, ΔD, was determined as the distance along the central line between ascending and descending aorta in the 'candy cane' image. FLOW (Medis, Lunden, the Netherlands) was used to measure flow in both ascending and descending aorta regions. For Δt assessment, a Matlab program was developed and flow upslope was calculated for each time point by taking flow values from neighboring time points using least squares means. The maximal upslope was selected as the largest upslope among all time series. We calculated PWV = ΔD/Δt and aortic compliance as AC = 1/(ρ*PWV^2^), where blood density ρ = 1057 kg/m. Linear regression was used to determine the relationships between AC and age.

## Results

**Figure 1:**
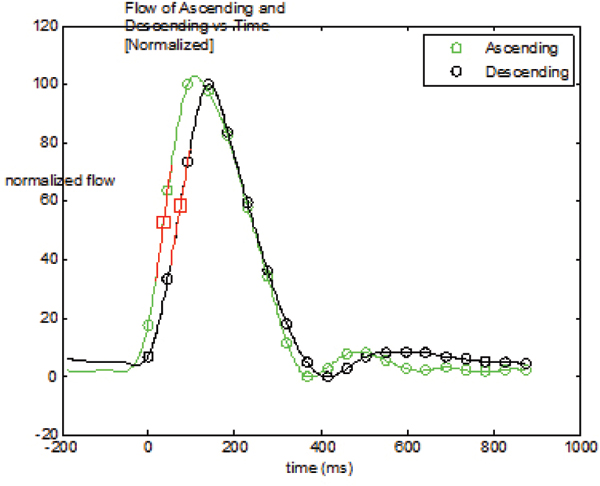
Figure 1

**Figure 2:**
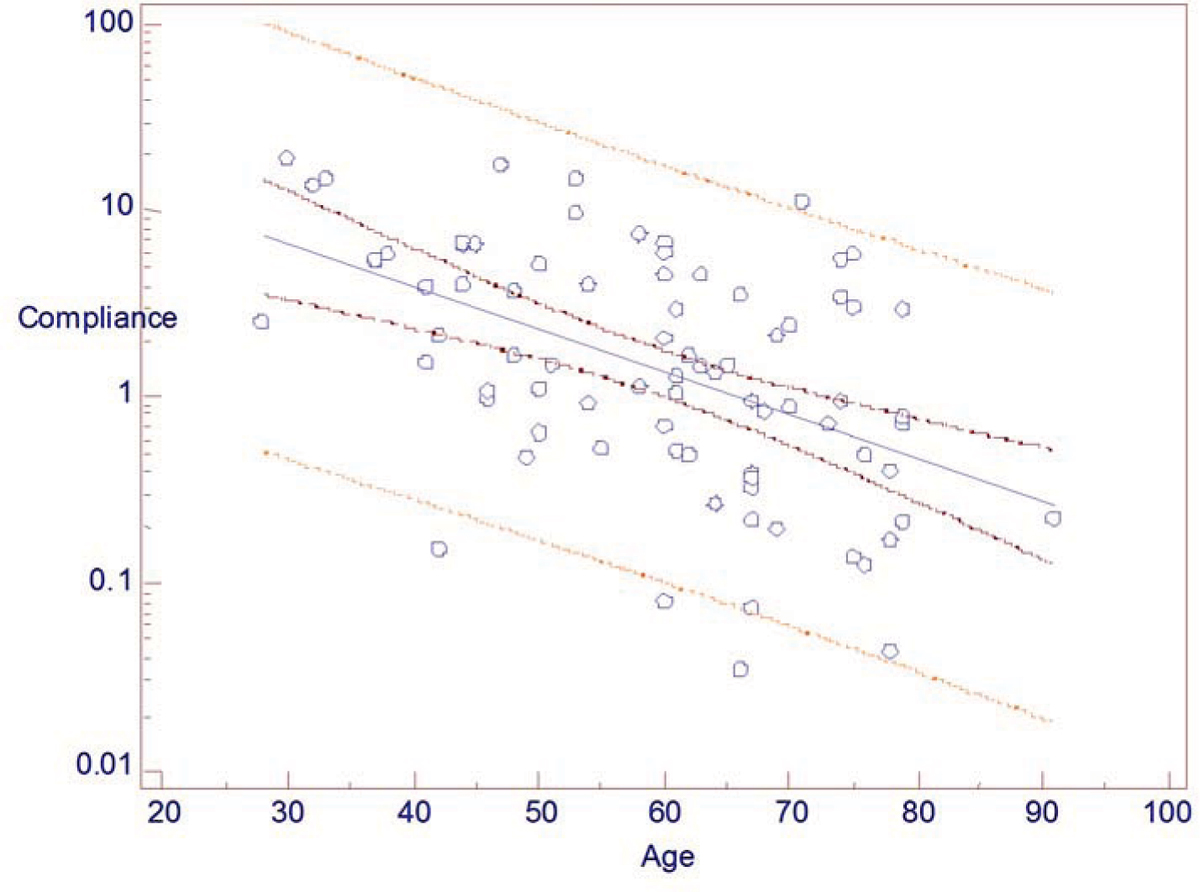
Figure 2

Illustrative ascending (green) & descending (black) aortic flow from ROIs in the PC images with maximal upslope occurred at the time marked with red squares and at an angle in the red lines are shown in Figure 1. The upslope algorithm worked well on all cases without any user interaction. The mean ± sd of PWV and AC were 11.1 ± 9.6 m/s and (3.2 ± 4.1)*10^-5^/Pa, respectively. Linear regression of logarithm AC vs. age had an R^2^ of 0.25 and p < 0.001, n = 80 (Figure 2). The regression showed stronger correlation in females, R^2^ = 0.34, p < 0.001, n = 46; while R^2^ = 0.13 and p = 0.04, n = 34 in males.

## Conclusion

Through-plane PC is an easy approach to evaluate aortic compliance in a single breath-hold. The aortic compliance results in normal volunteers using this imaging technique combined with the maximal upslope algorithm for Δt assessment showed a good correlation with age and has the potential to be an efficient clinical tool for assessment of vascular stiffness. Further comparison of this upslope approach to the other algorithms in different flow patterns is needed.

